# The effect of polypharmacy on healthcare services utilization in older adults with comorbidities: a retrospective cohort study

**DOI:** 10.1186/s12875-023-02070-0

**Published:** 2023-05-26

**Authors:** George Doumat, Darine Daher, Mira Itani, Lina Abdouni, Khalil El Asmar, Georges Assaf

**Affiliations:** 1 Faculty of Medicine, American University of Beirut Medical Center, Beirut, Lebanon; 2 Department of Family Medicine, Faculty of Medicine, American University of Beirut, Beirut, Lebanon; 3 Department of Epidemiology and Population Health, Faculty of Health Sciences, American University of Beirut, Beirut, Lebanon; 4 Division of Academic Internal Medicine & Geriatrics, The University of Illinois at Chicago, Chicago, USA

**Keywords:** Aged, Polypharmacy, Outcome Assessment, Health Care

## Abstract

**Background:**

Older adults are more prone to increasing comorbidities and polypharmacy. Polypharmacy is associated with inappropriate prescribing and an increased risk of adverse effects. This study examined the effect of polypharmacy in older adults on healthcare services utilization (HSU). It also explored the impact of different drug classes of polypharmacy including psychotropic, antihypertensive, and antidiabetic polypharmacy on HSU.

**Methods:**

This is a retrospective cohort study. Community-dwelling older adults aged ≥ 65 years were selected from the primary care patient cohort database of the ambulatory clinics of the Department of Family Medicine at the American University of Beirut Medical Center. Concomitant use of 5 or more prescription medications was considered polypharmacy. Demographics, Charlson Comorbidity index (CCI), and HSU outcomes, including the rate of all-cause emergency department (ED) visits, rate of all-cause hospitalization, rate of ED visits for pneumonia, rate of hospitalization for pneumonia, and mortality were collected. Binomial logistic regression models were used to predict the rates of HSU outcomes.

**Results:**

A total of 496 patients were analyzed. Comorbidities were present in all patients, with 22.8% (113) of patients having mild to moderate comorbidity and 77.2% (383) of patients having severe comorbidity. Patients with polypharmacy were more likely to have severe comorbidity compared to patients with no polypharmacy (72.3% vs. 27.7%, p = 0.001). Patients with polypharmacy were more likely to visit the ED for all causes as compared to patients without polypharmacy (40.6% vs. 31.4%, p = 0.05), and had a significantly higher rate of all-cause hospitalization (adjusted odds ratio aOR 1.66, 95 CI = 1.08–2.56, p = 0.022). Patients with psychotropic polypharmacy were more likely to be hospitalized due to pneumonia (crude odds ratio cOR 2.37, 95 CI = 1.03–5.46, p = 0.043), and to visit ED for Pneumonia (cOR 2.31, 95 CI = 1.00–5.31, p = 0.049). The association lost significance after adjustment.

**Conclusions:**

The increasing prevalence of polypharmacy amongst the geriatric population with comorbidity is associated with an increase in HSU outcomes. As such, frequent medication revisions in a holistic, multi-disciplinary approach are needed.

## Background

The advancements in modern medicine have led to an increase in life expectancy, and have subsequently created new challenges in treating older adults, mainly comorbidity [1]. Comorbidity is defined as the coexistence of 2 or more chronic conditions [1]. As the number of chronic conditions facing older adults increases, the management complexity for both the healthcare provider and the patient also increases. As such, comorbidity will naturally necessitate an increased number of medications to manage the different conditions. This is commonly referred to as polypharmacy, another challenge in geriatric patient management.

Although there is no clear definition in the literature, polypharmacy is commonly defined as the use of 5 or more medications at the same time [2]. Alternatively, a common qualitative definition of polypharmacy is the use of multiple medications concurrently by the same patient [2].

The prevalence of polypharmacy among older adults differs across countries, but a common pattern of increased prevalence was noted. In 2010, the prevalence of polypharmacy among adults in Scotland was 22.1%, as compared to 11.4% in 1995 [3]. A similar trend was noted in Sweden with polypharmacy prevalence reaching 44%, 50% of whom were aged 65 or more [4]. In the United States, the prevalence of polypharmacy among adults 65 years or older was 65.1% between 2009 and 2016 [5]. Similar patterns were observed in the Middle East, as the prevalence of polypharmacy in Saudi Arabia among patients 60 years or older reached 51.5% [6]. Moreover, a study found that 23.9% of adults above 65 years of age in rural areas in Lebanon were exposed to polypharmacy [7].

Although polypharmacy may be appropriate in some older adults [8], it has potential negative effects including adverse drug events (ADEs), increased healthcare services utilization (HSU) [9], cognitive impairment, reduced adherence, falls, and mortality [10]. A retrospective study documented that 10% of emergency department (ED) visits for adults aged 65 years or older can be attributed to ADEs [11]. Another retrospective study has shown that older veterans taking more than 5 medications were 4 times more likely to be hospitalized from ADEs [12]. Moreover, the risk of drug-drug interaction increases as the number of medications increases. A patient taking 5 to 9 medications has a 50% probability of drug interactions, and a patient taking more than 20 has a 100% probability [13]. ADEs will eventually lead to more HSU since it accounts for 6.5% of hospital admissions [14].

Furthermore, polypharmacy affects cognitive ability with the number of medications shown to be a risk factor for delirium [15]. A study of cognitive impairment in older adults showed that 33% and 54% of patients taking more than 5 and 10 medications, respectively, had cognitive impairment [16]. A meta-analysis examining the association between polypharmacy and mortality showed that polypharmacy is associated with higher mortality [17].

This study aims to examine the effect of polypharmacy in older adults on HSU outcomes as defined by the rate of all-cause ED visits, rate of all-cause hospitalization, rate of ED visits for pneumonia, rate of hospitalization for pneumonia, and mortality. A second aim is to explore the impact of different drug classes of polypharmacy including psychotropic, antihypertensive, and antidiabetic polypharmacy on HSU outcomes.

## Methods

### Study design, participants, and setting

This retrospective cohort study involved the secondary analysis of a primary care center’s dataset. The original dataset was created using a retrospective case-control design to examine the association between polypharmacy and dementia in community-dwelling older adults who are 65 years and older with dementia, and a control group attending the ambulatory clinics of the Department of Family Medicine at the American University of Beirut Medical Center (AUBMC), Beirut, Lebanon. The sample of this study consisted of all patients (n = 496) in the original primary care cohort of community-dwelling older adults ≥ 65 years of age. All data were retrieved from the medical records at the primary healthcare clinics. In the original dataset, the reference date indicated the date of diagnosis of the cases with dementia, and the selection date referred to the date of selection of controls without dementia. Cases were matched to three controls based on age and gender, and year of dementia diagnosis. In the current study, baseline data at the reference/selection date was collected and included age, gender, history of depression, medical comorbidities, smoking status, number, and type of prescription medications. The Charlson Comorbidity Index (CCI) [18] score and the Atherosclerotic Cardiovascular Disease (ASCVD) score were calculated based on data extracted from the reference/selection date. The ASCVD score was divided into two categories; low-intermediate risk if the score is < 20%, and high risk if ≥ 20% [19]. The CCI score was used as an indicator for comorbidity and calculated as a continuous variable and then divided into three CCI categories: mild- (≤ 2), moderate- (3 to 4), and severe risk (≥ 5). CCI was further collapsed into two categories; mild-moderate risk if the score is < 5, and high risk if ≥ 5 for the analysis [20]. The chronic diseases extracted and assessed were: congestive heart failure, peripheral vascular disease, cerebrovascular stroke, hemiplegia, dementia, chronic obstructive pulmonary disease, connective tissue disorders, peptic ulcer disease, liver disease, diabetes mellitus, renal disease, tumors, leukemia, lymphoma, and HIV/AIDS.

### Healthcare services utilization outcomes

HSU included 5 outcomes; the rate of all-cause ED visits, rate of all-cause hospitalization, rate of ED visits for pneumonia, rate of hospitalization for pneumonia, and mortality, all of which were collected within 3 years after the reference/selection date. ED visits and hospitalization for pneumonia were added as HSU outcomes since the prevalence of pneumonia increases with age and has a high short- and 1-year mortality rate among older adults [21]. HSU outcome rates were transformed to binary variables with ‘yes’ and ‘no’ options, and the ‘yes’ was defined as ≥ 2 episodes for all-cause ED visits and all-cause hospitalization, and ≥ 1 episode for ED visits for pneumonia, and hospitalization for pneumonia.

### Medication status

The list of prescription medications at the reference/selection date was collected. Polypharmacy is defined as the regular use of five or more medications daily [2]. All drugs recorded were coded according to the 2018 Anatomical Therapeutic Chemical (ATC) classification system developed by the WHO Collaborating Centre for Drug Statistics Methodology [22]. The dosage was not considered. Medications with different brand names and generics having the same ATC code were considered as one medicine. As-needed agents, topical agents, herbal and dietary supplements, and drugs for short-term use such as antibiotics and vaccinations were excluded.

Psychotropic, antihypertensive, and antidiabetic polypharmacy were defined as the concurrent use of two or more psychotropic, antihypertensive, and antidiabetic medications, respectively.

This definition is based on studies in the literature since there are no clear guidelines [23]. These specific classes of medications were associated with more adverse health outcomes in older adults compared to other medications [24–27]. Psychotropic medications include antidepressants, antiepileptics, antipsychotics, gabapentin, hypnotics, sedatives, anxiolytics, and mood stabilizers. The ATC codes of psychotropic medications include: N06AX21, N06AB10, N06AB03, N06AA02, N06AA14, N06AX11, N06AB05, N06AB06, N06AX16, N06AX26, N06AA09, N03AX18, N03AX14, N03AB02, N03AA03, N05AA01, N05AF01, N05AD01, N05AG02, N05AB04, N05AH04, N05AX08, N03AX12, N03AX16, N03AE01, N05CF04, N05BA06, N05CD08, N05CF02, N05BA12, N05BA08, N05BA02, N03AF01, N03AX09, N05AN01, N03AG01. The ATC code of antihypertensive medications include: C09AA02, C09AA03, C09AA04, C09AA06, C09AA05, C09AA10, C09AA09, C09AA01, C09CA06, C09CA04, C09CA01, C09CA08, C09CA07, C09CA03, C07AB03, C07AB07, C07AB09, C07AB02, C07AB12, C07AA05, C07AG02, C08CA13, C08CA05, C08DA01, C08CA01, C08EA02, C08DB01, C03DB01, C03CA02, C03DA04, C03CA01, C03AA03, C03BA, C03DA01, C03CA04, C03BA04, C01DA08, C01DA14, C01DX12, C01DX16, C01EB15. The ATC codes of the antidiabetic medications include: A10BF01, A10BA02, A10BH05, A10BH03, A10BH01, A10BH02, A10BJ02, A10AB01, A10AB05, A10AE06, A10AE05, A10AE04, A10AC04, A10BX02, A10BK01, A10BK03, A10BB01, A10BB09, A10BB12, A10BB08, A10BB01, A10BG03.

### Statistical analysis

The demographic, clinical characteristics and HSU outcomes of the study population were analyzed and reported as mean and standard deviation (SD) for continuous variables, and frequency and percentage for categorical variables. Bivariate analysis was performed for the demographics, clinical characteristics, and HSU outcomes using the Chi-squared test for categorical variables, and the independent T-test for continuous variables. Binomial logistic regression models were used to predict the rates of all-cause ED visits, all-cause hospitalization; ED visits due to pneumonia, hospitalization due to pneumonia, and mortality. Two separate models for HSU outcome were performed to avoid collinearity between polypharmacy and its drug classes. Model 1 was performed to examine the association between polypharmacy and HSU outcomes. It included polypharmacy and adjusted for the patients’ demographic and clinical characteristics that include dementia, age, gender, smoking status, depression, CCI, and ASCVD scores. Model 2 was performed to examine the association between the drug classes of polypharmacy (psychotropic, antihypertensive, and antidiabetic) and HSU outcomes. Model 2 was adjusted for the same demographic and clinical characteristics. Adjusting for dementia was necessary to minimize bias since the data was originally collected as a case-control design, with the cases defined by the diagnosis of dementia.

## Results

### Demographic and clinical characteristics of the study population

Table 1 summarizes the baseline characteristics of our sample of 496 patients. The mean age of our sample was 78.6 years old, of which 195 (39.3%) were females and 301 (60.7%) were males. Sixty-two (24.9%) patients had a low-intermediate risk ASCVD score and 187 (75.1%) had a high-risk ASCVD score. Comorbidities were present in all patients, with 22.8% (113) of patients having mild to moderate comorbidity (CCI < 5) and 77.2% (383) of patients having severe comorbidity (CCI ≥ 5).

**Fig. 1:**
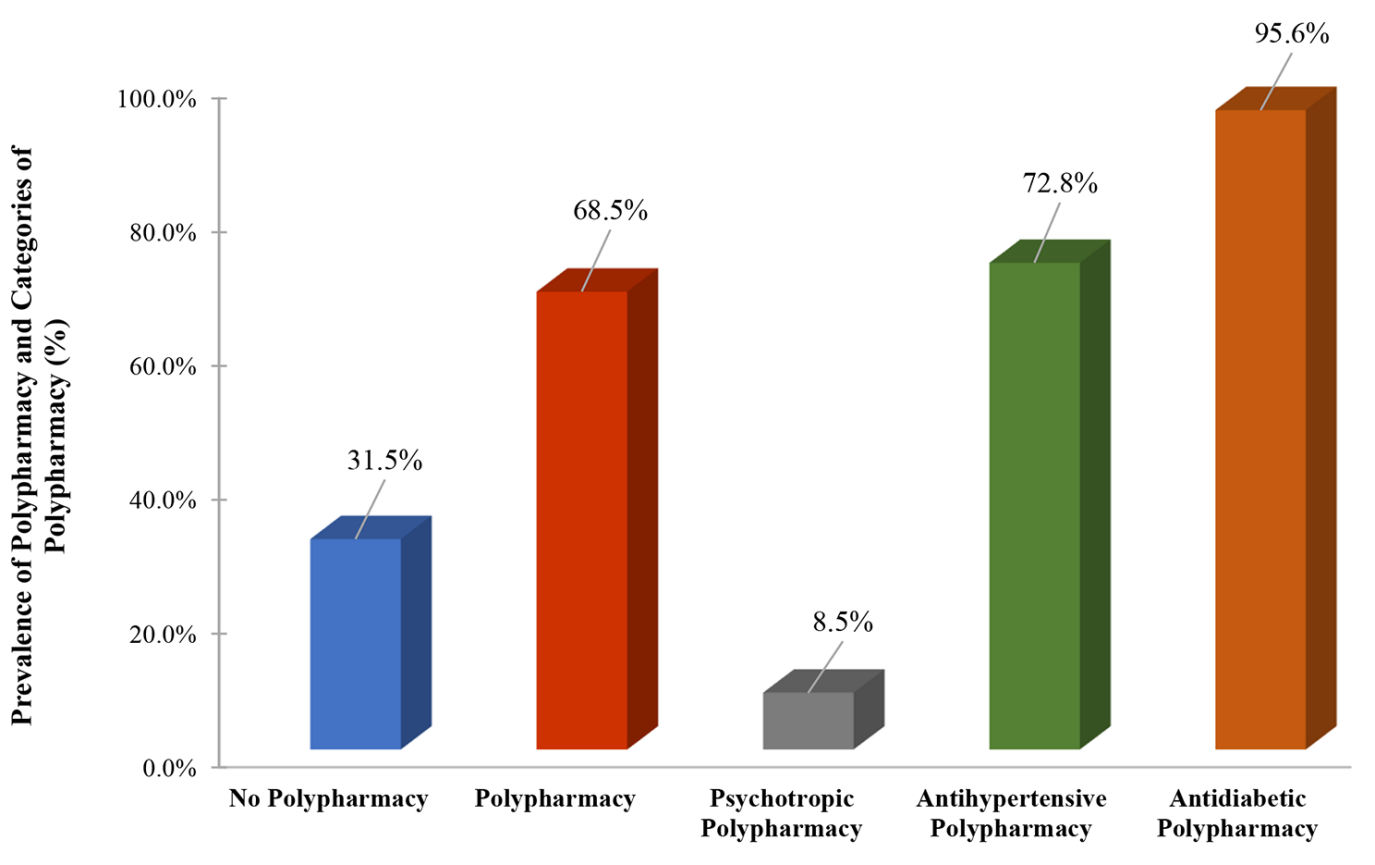
Prevalence of polypharmacy and drug classes of polypharmacy among the study population (n = 496)

**Fig. 2:**
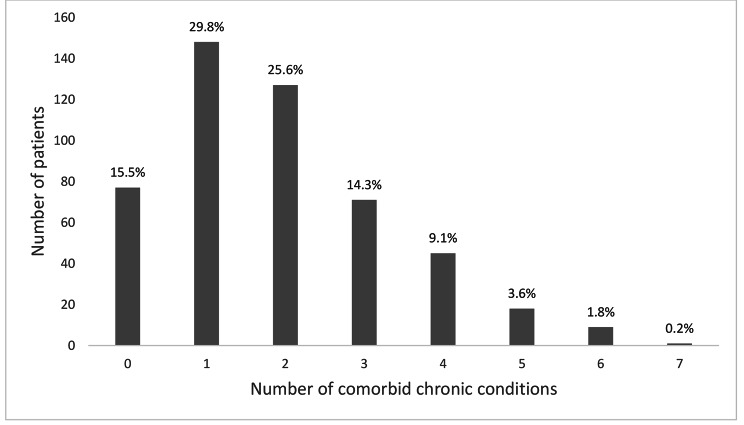
Proportion of comorbid medical conditions among the study population (n = 496)

One hundred and twenty-four (25%) patients had dementia. Overall, the prevalence of polypharmacy was 68.5%, 8.5% for psychotropic polypharmacy, 72.8% for antihypertensive polypharmacy, and 95.6% for antidiabetic polypharmacy (Fig. 1). The number of comorbid chronic conditions in our patient population is illustrated in Fig. 2.

**Table 1:** Descriptive statistics and bivariate analysis of patients’ characteristics and polypharmacy

Characteristic	No Polypharmacy (< 5)	Polypharmacy ( > = 5)	*P*-Value	Total
**N**	156 (31.5%)	340 (68.5%)		496
**Age** ^**a**^	78.2	78.8	0.942	78.6
**Gender** ^**b**^				
Male	68 (34.9%)	127 (65.1%)	0.187	301 (60.7%)
Female	88 (29.2%)	213 (70.8%)		195 (39.3%)
**Smoker (490)** ^**b**^				
Non-smoker	131 (30.9%)	293 (69.1%)	0.257	424 (86.5%)
Smoker	25 (37.9%)	41 (62.1%)		66 (13.5%)
**ASCVD Score** ^**b**^ **(249)**				
Low-intermediate risk	25 (40.3%)	37 (59.7%)	0.093	62 (24.9%)
High risk	54 (28.9%)	133 (71.1%)		187 (75.1%)
**Charlson Comorbidity Index (18)** ^**b**^				
Mild-Moderate	50 (44.2%)	63 (55.8%)	0.001*	113 (22.8%)
Severe	106 (27.7%)	277 (72.3%)		383 (77.2%)
**Dementia** ^**b**^				
Yes	38 (30.6%)	86 (69.4%)	0.823	124 (25%)
No	118 (31.7%)	254 (68.3%)		372 (75%)

^a^ Independent T−test

^b^ Chi−squared test

* Statistical significance is set at p < 0.05

### Bivariate analysis

Patients with polypharmacy had considerably more severe comorbidity (CCI ≥ 5) compared to the non-polypharmacy group (72.3% vs. 27.7%, p = 0.001), were more likely to visit the ED for all causes (40.6% vs. 31.4%, p = 0.05), and were more likely to be hospitalized (37.9% vs. 25.6%, p = 0.007). When mortality, ED visits for pneumonia, and hospitalization for pneumonia were compared between the polypharmacy and no polypharmacy groups, no statistically significant differences were found (Table 2).

**Table 2:** Descriptive statistics and bivariate analysis between polypharmacy and healthcare services utilization outcomes (3-year follow-up)

HSU Outcome		No Polypharmacy (< 5)	Polypharmacy ( > = 5)	*P*-Value	Total
**All-cause ED visits** ^a^					
Yes ( > = 2)		49 (31.4%)	138 (40.6%)	0.050	187 (37.7%)
No (< 2)		107 (68.6%)	202 (59.4%)		309 (62.3%)
**All-cause hospitalization** ^**a**^					
Yes ( > = 2)		40 (25.6%)	129 (37.9%)	0.007*	169 (34.1%)
No (< 2)		116 (74.4%)	211 (62.1%)		327 (65.9%)
**ED visits for pneumonia** ^**a**^					
Yes ( > = 1)		12 (7.7%)	38 (11.2%)	0.231	50 (10.1%)
No (0)		144 (92.3%)	302 (88.8%)		446 (89.9%)
**Hospitalization for pneumonia** ^**a**^					
Yes ( > = 1)		12 (7.7%)	37 (10.9%)	0.269	49 (9.9%)
No (0)		144 (92.3%)	303 (89.1%)		447 (90.1%)
**Mortality** ^**a**^					
Yes		28 (17.9%)	69 (20.4%)	0.653	97 (19.6%)
No		128 (82.1%)	270 (79.6%)		398 (80.4%)

^a^ Chi−squared test

* Statistical significance is set at p < 0.05

### Association between polypharmacy, drug classes of polypharmacy, and healthcare services utilization outcomes

In model 1, logistic regression was performed to examine the association between polypharmacy and HSU outcomes. Model 1 was adjusted for age, gender, smoking status, depression, dementia, CCI, and ASCVD scores. Patients with polypharmacy had a significantly higher rate of all-cause hospitalization (aOR 1.66, 95 CI = 1.08–2.56, P = 0.022) (Table 3). In model 2, logistic regression was performed to examine the association between drug classes of polypharmacy and HSU outcomes. Model 2 was adjusted for the same covariates as model 1. Patients with psychotropic polypharmacy were more likely to visit the ED (cOR 2.31, 95 CI = 1.00–5.31, P = 0.049) and to be hospitalized for pneumonia (cOR 2.37, 95 CI = 1.03–5.46, P = 0.043), however, this association lost significance when the model was adjusted. No significant association between antidiabetic and antihypertensive polypharmacy and HSU outcomes was found (Table 4).

**Table 3:** Binomial logistic regression for the association between polypharmacy and healthcare services utilization

HSU Outcome	Odds Ratio (95% CI) *P*-Value	
	Unadjusted Odds Ratio	Adjusted Odds Ratio*
**All-cause ED visits**	1.49 (0.99–2.23) 0.051	1.34 (0.89–2.03) 0.166
**All-cause hospitalization**	**1.77 (1.16–2.70)** 0.008**	**1.66 (1.08–2.56)** 0.022**
**ED visits for pneumonia**	1.51 (0.77–2.97) 0.234	1.35 (0.67–2.69) 0.400
**Hospitalization for pneumonia**	1.47 (0.74–2.89) 0.271	1.33 (0.66–2.66) 0.427
**Mortality**	1.16 (0.72–1.89) 0.541	1.05 (0.63–1.75) 0.846

* Adjusted for dementia, age, gender, depression, smoking, CCI, and ASCVD score

** Statistical significance is set at p < 0.05

**Table 4:** Binomial logistic regression for the association between polypharmacy classes and healthcare services utilization

HSU Outcome	Odds Ratio (95% CI) *P*-Value	
	Unadjusted Odds Ratio	Adjusted Odds Ratio*
**Psychotropic Polypharmacy**		
All-cause ED visits	1.27 (0.67–2.40) 0.472	1.04 (0.52–2.07) 0.913
All-cause hospitalization	1.67 (0.88–3.17) 0.114	1.38 (0.70–2.73) 0.357
ED visits for pneumonia	**2.31 (1.00–5.31)** 0.049**	1.63 (0.65–4.08) 0.301
Hospitalization for pneumonia	**2.37 (1.03–5.46)** 0.043**	1.74 (0.69–4.38) 0.239
Mortality	1.74 (0.85–3.53) 0.128	1.37 (0.63–2.99) 0.431
**Antihypertensive Polypharmacy**		
All-cause ED visits	0.88 (0.58–1.31) 0.518	0.86 (0.56–1.33) 0.495
All-cause hospitalization	0.80 (0.53–1.21) 0.287	0.79 (0.51–1.23) 0.293
ED visits for pneumonia	0.70 (0.38–1.30) 0.258	0.72 (0.37–1.42) 0.342
Hospitalization for pneumonia	0.75 (0.40–1.41) 0.369	0.78 (0.39–1.55) 0.474
Mortality	0.71 (0.44–1.14) 0.156	0.82 (0.49–1.38) 0.459
**Antidiabetic Polypharmacy**		
All-cause ED visits	1.47 (0.63–3.42) 0.376	1.76 (0.73–4.29) 0.210
All-cause hospitalization	1.04 (0.46–2.36) 0.934	1.30 (0.55–3.08) 0.551
ED visits for pneumonia	1.43 (0.33–6.20) 0.637	1.48 (0.32–6.84) 0.612
Hospitalization for pneumonia	1.39 (0.32–6.06) 0.659	1.42 (0.30–6.56) 0.650
Mortality	0.68 (0.28–1.65) 0.393	0.63 (0.24–1.64) 0.345

* Adjusted for dementia, age, gender, depression, smoking, CCI, and ASCVD score

** Statistical significance is set at p < 0.05

## Discussion

The present study examined the effect of polypharmacy on HSU outcomes in older adults with comorbidities. It also shed light on the impact of different drug classes of polypharmacy, including psychotropic, antihypertensive, and antidiabetic polypharmacy on HSU outcomes.

We report a high prevalence of polypharmacy (68.5%) in our study sample, which is consistent with prior studies that found comparable rates [7–9]. Our findings revealed a lower prevalence rate of psychotropic polypharmacy (8.5%) compared to previous research (18% and 44.9%) [29, 30]. These disparities could be attributed to variations in study design and settings. To the best of our knowledge, there have been no studies that describe the prevalence of antidiabetic and antihypertensive polypharmacy. Patients with polypharmacy had higher comorbidity scores, which is in line with previous research indicating that people with multiple chronic conditions are more likely to have polypharmacy [28, 29].

We found a significant association between polypharmacy and all-cause hospitalization, consistent with previous studies [30–32]. The increase in hospital admissions can be attributed to the adverse effects of medications. This is further supported by evidence suggesting that as the number of prescribed medications increases, so does the likelihood of prescribing error, high-risk prescribing, and ADEs [33]. Despite controlling for comorbidities, it is important to note that comorbidity indices quantify the burden of coexisting medical conditions and often do not reflect the clinical complexity of the older patient or the severity of chronic diseases, which may partially explain the observed association between polypharmacy and hospitalization [34]. We did not find an association between polypharmacy and mortality. Although some studies [17, 32] demonstrated an association between polypharmacy and increased mortality in older adults, others have been inconsistent and failed to find these associations [35]. The causality of the relationship remains unclear and must be carefully considered [32].

Psychotropic polypharmacy was found to increase the likelihood of ED visits for pneumonia, and hospitalization for pneumonia. However, after adjustment, the association lost significance. No association was found between antihypertensive and antidiabetic polypharmacy and HSU outcomes. Our findings are supported by recent evidence indicating that the use of psychotropic drugs (antipsychotics, benzodiazepine [BZD], and benzodiazepine-related drugs) is associated with an increased risk of pneumonia in older adults aged ≥ 65 years [36, 37]. This relationship increased in strength as age increased, which also supports our explanation in the setting of older adults [36]. First, the extrapyramidal side effects of antipsychotics, in particular tardive dyskinesia, increase the likelihood of aspiration because of involuntary buccolingual movements and oropharyngeal dysphagia [38]. Second, the histamine receptor-blocking ability of antipsychotics, coupled with the age-related changes of the airway, causes patients to inadequately handle secretions, which can lead to aspiration [39].

Moreover, a recent systematic review and meta-analysis found that older adults aged ≥ 65 years who were exposed to BZD had an increased risk of pneumonia [40]. The sedative effect induced by BZDs in older adults may lead to an increased risk of aspiration pneumonia [41, 42]. In addition, BZD exposure has been associated with an increased risk of gastroesophageal reflux disease (GERD) in older adults, which may be related to the relaxation of the esophageal sphincter, possibly leading to aspiration [41, 42]. Animal studies showed that BZDs increase vulnerability to infection by activating specific Gamma-aminobutyric acid (GABA) receptors on immune cells [43].

Studies on the association between antidepressant use and the development of pneumonia yielded mixed results. When controlling for confounders, it was found that antidepressants were not associated with an increased risk of pneumonia [44]. However, a strong body of evidence suggests that antidepressants increase the risk of pneumonia through anticholinergic properties [45] and its sedative effects [46].

The association between psychotropic polypharmacy and pneumonia should be explored in light of the indications and diagnoses associated with this class of medications. This was evident in our results, as the association between psychotropic polypharmacy and pneumonia, both ED visits and admission lost significance after adjusting for potential confounders. One explanation can stem from the well-documented association between dementia and the increased risk of pneumonia-related healthcare utilization [47]. Moreover, psychiatric disorders like depression, schizophrenia, and bipolar disorder, are associated with a higher risk of having pneumonia compared to the general population [48, 49]. Thus the association of psychotropic polypharmacy and pneumonia-related healthcare utilization should be considered with the different cofounders in mind.

Despite the lack of evidence on the association between regular medication review and prevention of adverse clinical outcomes, such as hospitalization and mortality [50], our findings encourage regular medication review as part of clinical encounters to reduce polypharmacy and inappropriate prescribing and to identify potential drug-drug interactions. It is crucial to focus on “high-risk” medication classes, their indications, and their risk-benefit ratios. One solution is adopting a multidisciplinary approach when caring for older adults, which involves the input of physicians, clinical pharmacists, and other healthcare personnel. This collaborative approach improves medication appropriateness by alerting the healthcare provider about polypharmacy and informing the patient about potential side effects and warning signs [51]. Another solution would be to use the American Geriatrics Society Beers Criteria® (AGS Beers Criteria®) for Potentially Inappropriate Medication (PIM) use in older adults as a screening tool to taper down or discontinue inappropriate medications [52].

### Strengths and limitations

To the best of our knowledge, this is the first study in a primary care setting that explored the association between polypharmacy and drug classes of polypharmacy and HSU outcomes. Nevertheless, we acknowledge that there are limitations to our study. First, cases and controls were both selected from the same clinic and hospital, which increases selection bias, and make the data less generalizable to the population. Second, this study was conducted in a tertiary medical center, which makes it susceptible to referral bias. The results can be prone to assessor bias in making the diagnosis of dementia. Since the cohort was not randomized but selected as cases and controls, differences in the character of each group can introduce confounders in the results. The cohort was taken from a dataset originally collected as a case-control study assessing the effect of polypharmacy on dementia; however, adjusting for dementia and other potential confounders in the models decreased the chance of bias. Given that our study was retrospective chart review research, incomplete or missing documentation may have occurred. Our study did not investigate the impact of medication interactions, either within the same class or across different classes, or medication adherence on healthcare-associated outcomes. Other outcomes could be explored in future studies, such as falls and related ED visits or hospitalization to further our understanding of the prognostic impact of polypharmacy. Future studies are also needed to examine the effect of drug interactions and medication adherence on healthcare utilization and outcomes.

## Conclusion

The advancements in modern medicine have brought about an ever-growing aging population, which presents a unique set of challenges. Comorbidity and consequently polypharmacy warrants particular attention due to potential inappropriate prescribing and consequent adverse events in this age group. This study demonstrated an association between polypharmacy and all-cause hospitalization in older adults. It further highlighted the effect of psychotropic polypharmacy on ED visits for pneumonia, and hospitalization for pneumonia. These findings indicate the need for frequent medication revisions among older adults, in a holistic, multi-disciplinary approach.

## Data Availability

The datasets generated and analyzed during the current study are not publicly available to protect patients’ confidentiality but are available from the corresponding author upon reasonable request.

## Abbreviations

AUBMC: American University of Beirut Medical Center
HSU: Healthcare Services Utilization
ED: Emergency Department
CCI: Charlson Comorbidity Index
ASCVD: Atherosclerotic Cardiovascular Disease
ADEs: Adverse Drug Events
ATC: Anatomical Therapeutic Chemical
SD: Standard deviation
BZD: benzodiazepine
GERD: Gastroesophageal reflux disease
GABA: Gamma-aminobutyric acid
AGS: American Geriatrics Society
PIM: Potentially Inappropriate Medication.
